# Vitamin K Antagonist Use and Risk for Intracranial Carotid Artery Calcification in Patients With Intracerebral Hemorrhage

**DOI:** 10.3389/fneur.2019.01278

**Published:** 2019-12-20

**Authors:** Michaël T. J. Peeters, Rik Houben, Alida A. Postma, Robert J. van Oostenbrugge, Leon J. Schurgers, Julie Staals

**Affiliations:** ^1^Department of Neurology and Cardiovascular Research Institute Maastricht, Maastricht University Medical Center, Maastricht, Netherlands; ^2^Department of Radiology and Nuclear Medicine, Maastricht University Medical Center, Maastricht, Netherlands; ^3^Department of Biochemistry, Cardiovascular Research Institute Maastricht, Maastricht, Netherlands

**Keywords:** intracranial carotid artery calcification, vitamin K antagonist, intracerebral hemorrhage, vascular calcification, computed tomography

## Abstract

**Background:** Intracranial carotid artery calcification (ICAC) on computed tomography (CT) is a marker of atherosclerosis and an independent predictor of vascular events including stroke. While vitamin K antagonists (VKAs) are used to prevent embolic stroke, they have been shown to increase levels of both coronary and extracoronary artery calcification. This has not been studied for (intracranial) carotid arteries. The aim of this study is to investigate the association between VKA use and degree of ICAC. We tested our hypothesis in a cohort of patients with nontraumatic intracerebral hemorrhage (ICH) of which a substantial part used VKAs.

**Materials and Methods:** We retrospectively semiquantified ICAC on brain unenhanced CT of consecutive adult patients with nontraumatic ICH. Assessment was performed blinded to clinical characteristics and status of VKA use. We used a 5-point visual scale and dichotomized degree of ICAC in low and high degree. Patient demographics, VKA use, duration of VKA treatment, as well as known risk factors for intracranial calcification were collected. Univariable and multivariable logistic regression analyses were performed to investigate the association between ICAC and VKA use.

**Results:** Three hundred and seventy-six nontraumatic ICH patients were included of whom 77 were using VKAs (20.5%) with a median treatment duration of 35 months. Any degree of ICAC was detected in 289 patients (76.9%). Univariable analysis showed that a high degree of ICAC was significantly associated with older age [odds ratio (OR), 1.06, 95% confidence interval (CI), 1.03–1.08], hypertension (OR, 2.14; 95% CI, 1.27–3.62), diabetes mellitus (OR, 2.38; 95% CI, 1.27–4.49), and the use of VKAs (OR, 1.84; 95% CI, 1.06–3.20). In multivariable regression analysis, only older age was significantly associated with a higher degree of ICAC (OR, 1.05; 95% CI, 1.02–1.08), while VKA use was not (OR, 1.22; 95% CI, 0.67–2.24).

**Conclusions:** Our findings do not support VKA use as an independent risk factor for higher ICAC degree in patients with ICH. We could not confirm the concerns about VKA use and intracranial carotid vascular calcification. We suggest further research in other cohorts with VKA users such as patients with ischemic stroke and atrial fibrillation.

## Introduction

Vascular calcification is involved in the process of atherosclerosis and occurs in up to 90% of atherosclerotic lesions (1). Traditional cardiovascular risk factors such as aging, diabetes, hypercholesterolemia, smoking, and male sex are associated with increased coronary artery calcification, intracranial carotid artery calcification (ICAC), as well as peripheral arterial calcification (2–5).

Vitamin K antagonists (VKAs) are linked to higher levels of coronary artery calcification and extracoronary vascular calcification (6, 7). Animal experiments revealed that VKA use decreases activity of matrix GIa protein (MGP). MGP is a vitamin K-dependent protein which prevents soft tissue calcification, and decreased MGP activity may therefore lead to increased vascular calcification (8, 9). Despite extensive data which indicate that VKA use increases risk of vascular calcification, this has not been studied for (intracranial) carotid arteries.

The association between ICAC and VKA use could be of clinical importance. While VKA is used to prevent embolic stroke, ICAC is related to a higher atherosclerosis-related ischemic stroke risk (10–12). The Rotterdam study, a prospective cohort population study investigating risk factors of cardiovascular and neurological disease, showed that presence and severity of ICAC is a risk factor for ischemic stroke, independent of conventional risk factors and atherosclerosis in other vascular beds (11). Unenhanced brain CT scan is the most accessible and direct method to evaluate ICAC, and several methods to evaluate severity of ICAC on unenhanced CT imaging have been described (13, 14). We hypothesize that VKA use is associated with higher visual ICAC scores on unenhanced brain CT. We tested our hypothesis in a cohort of patients with nontraumatic intracerebral hemorrhage (ICH) of which a substantial part used VKAs.

## Materials and Methods

### Patient Selection

We conducted a retrospective cohort study. All adults (aged 18 years or older) with an imaging confirmed nontraumatic ICH admitted to the Maastricht University Medical Center, Netherlands, between January 1st 2004 and December 31st 2009, were included. In agreement with Dutch legislation, approval of medical ethical committees was waived because it was a retrospective chart study.

Patients with traumatic ICH, nonparenchymatous hemorrhage (e.g., subdural, epidural, subarachnoid, and isolated intraventricular hemorrhage), ischemic stroke with hemorrhagic transformation, or hemorrhage associated with a brain tumor were excluded as well as recurrent ICH cases within the same research period. Patients with non-accessible charts and/or brain scans were also excluded from analysis.

### Patient Characteristics and Definitions

We recorded age, sex, use of VKA, and duration of VKA therapy at the time of presentation. Known cardiovascular risk factors associated with ICAC (hypertension, diabetes mellitus, hyperlipidemia, current smoking) were collected as well.

Hypertension was defined as known hypertension from medical history or use of blood pressure lowering medication. Patients were considered diabetic if fasting plasma glucose was ≥7.8 mmol/l or if they were known diabetic [with or without use of (oral) antidiabetic drugs]. Hypercholesterolemia was defined as a total cholesterol ≥6.5 mmol/l or if subjects were using cholesterol lowering agents.

### Assessment of ICAC

Unenhanced cranial CT was performed in all patients according to standard practice on a 16-slice multidetector CT scanner. The slice thickness of most performed cranial CT investigations was 4–5 mm, with a minority of CT investigations consisting of 1.25-mm slices (*n* = 27, 7.2%). One reviewer blinded to clinical data and VKA status rated all scans, after first reaching substantial intrarater (weighted Cohen's kappa of 0.67 for right and 0.62 for left carotid artery) and moderate interrater agreement (weighted Cohen's kappa of 0.57 for right and 0.59 for left carotid artery; disagreements were solved by consensus) on 50 CT scans rated by two researchers.

**Figure 1:**
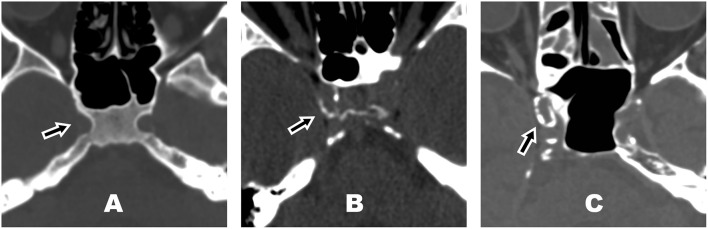
Examples of different degrees of intracranial carotid artery calcification in the right carotid artery (arrow) on unenhanced bone-window computed tomography (CT) image. **(A)** Intracranial carotid artery calcification (ICAC) score = 0 (no calcification), **(B)** ICAC score = 2 (mild, 10–50% of the vessel circumference constituted of calcification), **(C)** ICAC score = 4 (severe, >90% of the vessel circumference constituted of calcification).

We quantified the amount of ICAC using a 5-point visual scale, which is an adjusted version of the method as previously described by Chen et al. (13). In short, foci of calcification were scored from the skull base to the circle of Willis of both internal carotid arteries. The degree of calcification of each artery was visually rated and defined as follows (Figure 1): 0 = no calcification, 1 = minimal (1–10% of the vessel circumference constituted of calcification), 2 = mild (10–50%), 3 = moderate (50–90%), or 4 = severe (>90%). Each internal carotid artery was evaluated in the plane where it had the most circular cross-section for the thickness and extent of calcifications, respectively.

For statistical analyses, we dichotomized the degree of ICAC. We took the highest ICAC score of either left or right internal carotid artery and defined high ICAC degree as an ICAC score of ≥3 (≥50% of vessel circumference constituted of calcification). The interrater agreement for the applied dichotomized scale proved to be substantial (Cohen's kappa = 0.68).

### Statistical Analysis

Statistical analysis was performed using SPSS (SPSS version 25.0, IBM Corp., Amonk, NY, USA). To test for differences between groups (VKA users vs. non-VKA users), chi-square test for categorical data, independent *t*-test for normally distributed data, and Mann–Whitney test and Kruskal–Wallis test for non-normally distributed data were used.

Associations between degree of ICAC (dependent variable) and VKA use, patient demographics, and cardiovascular risk factors hypertension, diabetes, and hypercholesterolemia (independent variables) were tested with univariable and multivariable logistic regression analysis. Statistical significance was set at *p* ≤ 0.05.

## Results

We recorded 401 consecutive patients with nontraumatic ICH between 2004 and 2009. Of those, 25 (6.2%) were excluded due to various reasons: 7 patients only had MR imaging, CT imaging was inaccessible in 17 patients, and one patients' CT imaging quality was too low due to movement artifacts. There were no significant differences regarding demographics, VKA use, and cardiovascular risk factors between included and excluded patients. We included 376 patients for analyses. Patient characteristics are presented in Table 1. Mean age was 71.2 years (SD, 13.5), 200 patients were male (53.2%), and 77 patients were using VKAs (20.5%).

**Table 1:** Patient characteristics.

**Variable**	***N* (% of total)**	**Total group *N* = 376**	**VKA users *N* = 77 (20.5%)**	**No VKA users *N* = 299 (79.5%)**	***P*-value**
Mean age, years (SD)	376 (100)	71.2 (SD 13.5)	77.7 (SD 9.1)	69.6 (SD 14.0)	<0.001
Sex, male, *n* (%)	376 (100)	200 (53.3)	42 (54.5)	158 (52.8)	0.889
Hypertension, *n* (%)	372 (99.0)	220 (59.1)	64 (83.1)	156 (52.9)	<0.001
Smoking, *n* (%)	238 (63.3)	63 (26.5)	10 (20.4)	53 (28.0)	0.369
Hypercholesterolemia, *n* (%)	359 (95.5)	146 (40.7)	36 (48)	110 (38.7)	0.186
Diabetes mellitus, *n* (%)	373 (99.2)	49 (13.1)	14 (18.2)	35 (11.8)	0.200
VKA duration of treatment in months, median (IQR)^*^	–	–	35 (16.5–69.5)	–	–
High ICAC score, *n* (%)	376 (100)	87 (23.1)	25 (32.5)	62 (20.7)	0.043

*VKA, vitamin K antagonist; ICAC, intracranial carotid artery calcification; SD, standard deviation; IQR, interquartile range*.

* *Missing in four patients*.

### Intracranial Carotid Artery Calcification and Its Predictors

Any ICAC was present in 289 patients (76.9%). Eighty-seven patients (23.1%) had a high ICAC score. Table 1 shows the characteristics of patients using VKAs and those who did not use VKAs. Compared to patients not using VKAs, VKA users were significantly older and more often had hypertension as well as a high ICAC score.

Table 2 shows the associations between a high ICAC score and sex, age, traditional cardiovascular risk factors, as well as VKA use. Current smoking was excluded from logistic regression analysis because of missing data in 37% of included patients. In univariable regression analysis, a high ICAC score was associated with VKA use [odds ratio (OR), 1.84; 95% confidence interval (CI), 1.06–3.20]. Age, hypertension, and diabetes were also associated with a high ICAC score. In multivariable logistic regression analysis including age, sex, hypertension, hypercholesterolemia, and diabetes, there was no independent association of VKA use with a high ICAC score (OR, 1.22; 95% CI, 0.67–2.24). Of all covariables, only age was found to be an independent predictor for a high ICAC score (OR, 1.05; 95% CI, 1.02–1.08, per year).

**Table 2:** Univariable and multivariable logistic regression analysis for variables associated with ICAC.

	**Univariable analysis OR**	**95% CI**	***P*-value**	**Multivariable analysis OR**	**95% CI**	***P*-value**
Age (per year)	1.06	1.03–1.08	<0.001	1.05	1.02–1.08	<0.001
Sex, male	0.65	0.40–1.05	0.076	0.96	0.56–1.66	0.887
Hypertension	2.14	1.27–3.62	0.004	1.43	0.79–2.60	0.240
Hypercholesterolemia	1.36	0.83–2.23	0.220	1.11	0.64–1.92	0.702
Diabetes mellitus	2.38	1.27– 4.49	0.007	1.85	0.92–3.70	0.083
VKA use	1.84	1.06–3.20	0.031	1.22	0.67–2.24	0.512

*OR, odds ratio; CI, confidence interval*.

Duration of VKA treatment could be retrieved in 74 of 77 VKA users (93.6%) with median treatment duration of 35 months (interquartile range, 16.5–69.5 months). In VKA users, we could not show an association between duration of VKA treatment and a high ICAC score, neither in univariable nor in multivariable regression analysis (OR, 0.99).

## Discussion

In this study in 376 consecutive patients with nontraumatic ICH, we found no independent association between VKA use and higher degree of ICAC. Only age was found to be independently associated with higher degree of ICAC in ICH patients.

The first evidence of association between VKAs and vascular calcification in humans came from aortic valves obtained after routine cardiac replacement. Histopathological inspection showed a more than 2-fold higher calcification grade in VKA-treated patients when compared to non-treated patients (15). Radiological confirmation came from observational CT studies, which showed that long-term VKA use is associated with increased valvular and coronary calcification in patients with aortic valve disease as well as in patients with atrial fibrillation (7, 9, 16). A higher degree of vascular calcification was also found in peripheral arteries in VKA users when compared to matched control patients (17, 18).

We found any ICAC to be present in 76.9% of all ICH patients. A single other study in a cohort of 343 ICH patients due to cerebral small vessel disease found ICAC to be present in 74%. Although these results may seem comparable, different CT(A) techniques as well as definitions of presence of ICAC were used (19). Prevalence rates of ICAC in ischemic stroke populations are generally found to be comparable or slightly higher (72.0–92.6%) (10, 20).

We found no independent association between VKA use and ICAC. Apart from methodologic limitations, this could be due to study population. As ICH is mostly due to small vessel disease, our patients have a predilection to suffer from small vessel disease and might be less susceptible for developing severe large vessel atherosclerotic disease. Furthermore, the median duration of VKA treatment could be too short to show an association between VKA use and ICAC. Both animal experiments in rats as well as retrospective studies in humans indicate that longer VKA treatment as well as higher VKA doses increase tissue calcification, peripheral arterial calcification, as well as coronary plaque calcification (21, 22). A single retrospective cohort study in humans showed VKA treatment for more than 5 years to be an independent predictor of progressing peripheral arterial calcification, whereas a VKA treatment duration of 1–5 years was not (18). In our cohort, the median duration of VKA treatment was approximately 3 years, with only 19 patients using VKAs for more than 5 years (24.6%). Furthermore, we had no information on therapy compliance and average target international normalized ratio.

The main study strength is that a substantial part of our ICH cohort used VKA (20.5%), which makes it a suitable cohort to investigate the relationship between VKA use and ICAC. As all patients who present with acute stroke in our hospital get a routine diagnostic unenhanced CT scan, selection bias is largely excluded.

The main limitation of this study is the retrospective design. Although most important vascular risk factors could be reliably and fully obtained, there is missing data on current smoking as well as other predictors of ICAC such as kidney disease, alcohol intake, previous cardiac disease, and raised white blood cell count (23). The visual ICAC assessment was limited by the slice thickness on CT imaging. Only in a minority of cases thin slice (1.25 mm) CT was available (*n* = 27, 7.2%). All other CT investigations consisted of thicker 4- or 5-mm slices, which could influence the accuracy of ICAC assessment due to increased risk of partial volume effect. This was also reflected in the interrater agreement of our ICAC assessment, which was only substantial. A drawback of ICAC assessment in general is the lack of a consistently used and validated method of ICAC evaluation, with many different visual and (semi-) quantitative methods being used in the literature. Nevertheless, it has been shown that a 4- to 5-point visual degree scale, as used in our study, correlates highly with quantitative ICAC measures (14). Likewise, the imaging tools used to assess ICAC vary with previous studies reporting the use of conventional thick slice non-contrast CT, thin slice non-contrast CT, CT angiography, MR angiography, or even *in vivo* measurements for investigating ICAC burden. A single study which compared non-contrast thick slice CT to CT angiography showed the first to be more sensitive for detection of calcifications when compared to angiography. Overall agreement found in this study between CT and CT angiography-based ICAC ratings was good (20). We therefore believe that the currently used method and data derived from non-enhanced thick slice CT scan is sufficient for testing our hypothesis.

## Conclusions

Our results do not support VKA use as an independent risk factor for higher degree of ICAC in ICH patients. This study could not confirm the concerns about VKA use and intracranial carotid vascular calcification. More prospective research in other cohorts such as ischemic stroke and atrial fibrillation is needed to further explore the relationship between VKA use and ICAC.

## Data Availability Statement

The datasets generated for this study are available on request to the corresponding author.

## Ethics Statement

Ethical review and approval was not required for the study on human participants in accordance with the local legislation and institutional requirements. Written informed consent for participation was not required for this study in accordance with the national legislation and the institutional requirements.

## Author Contributions

JS, RH, AP, RO, and LS contributed to the conception and design of the study. RH, JS, and AP collected the data. RH, JS, and MP organized the database. MP and JS performed the statistical analysis and wrote the first draft of the manuscript. All authors contributed to manuscript revision, read, and approved the submitted version.

### Conflict of Interest

AP was speaker for Bayer (2017) as well as Siemens (2018). The remaining authors declare that the research was conducted in the absence of any commercial or financial relationships that could be construed as a potential conflict of interest.
